# Placental HSD2 Expression and Activity Is Unaffected by Maternal Protein Consumption or Gender in C57BL/6 Mice

**DOI:** 10.1155/2013/867938

**Published:** 2013-05-28

**Authors:** Mark R. Garbrecht, Fred S. Lamb

**Affiliations:** ^1^Department of Biology, Winona State University, 234 Pasteur Hall, P.O. Box 5838, Winona, MN 55987, USA; ^2^Department of Pediatrics, 5111 DOT, Vanderbilt Children's Hospital, Vanderbilt University, Nashville, TN 37232, USA

## Abstract

The placenta acts as a physiological barrier, preventing the transfer of maternal glucocorticoids to the developing fetus. This is accomplished via the oxidation, and subsequent inactivation, of endogenous glucocorticoids by the 11-**β** hydroxysteroid dehydrogenase type 2 enzyme (HSD2). Maternal protein restriction during pregnancy has been shown to result in a decrease in placental HSD2 expression and fetal glucocorticoid overexposure, especially late in gestation, resulting in low birth weight and “fetal programming” of the offspring. This dietary intervention impairs fetal growth and cardiovascular function in adult C57BL/6 offspring, but the impact on placental HSD2 has not been defined. The goal of the current study was to examine the effects of a maternal low-protein diet (18% versus 9% protein) on placental HSD2 gene expression and enzyme activity in mice during late gestation. In contrast to previous studies in rats, a maternal low-protein diet did not affect HSD2 protein or enzyme activity levels in the placentas of C57BL/6 mice and this was irrespective of the gender of the offspring. These data suggest that the effects of maternal protein restriction on adult phenotypes in C57BL/6 mice depend upon a mechanism that may be independent of placental HSD2 or possibly occurs earlier in gestation.

## 1. Introduction

A wealth of evidence demonstrates that an individual's risk of developing adult onset cardiovascular disease and the metabolic syndrome can be “programmed” by events occurring *in utero*. Studies aimed at gaining a better understanding of the fetal origins of adult-onset diseases have provided evidence that maternal diet during gestation can have a profound impact on the incidence of these conditions. Maternal dietary protein restriction during pregnancy has been linked to decreased birth weight and subsequent hypertension, coronary artery disease, obesity, and diabetes in adulthood [1, 2].

Glucocorticoids have been implicated as playing a fundamental role in the fetal programming process. Although required for the normal development and maturation of a number of organ systems, glucocorticoid overexposure is known to have a negative impact on normal growth and development and is associated with low birth weight, hypertension, and altered reactivity of the hypothalamic-pituitary-adrenal axis [3, 4]. The placenta acts as a physiological barrier, preventing the transfer of maternal glucocorticoids to the fetus primarily through the action of the microsomal enzyme, 11-*β* hydroxysteroid dehydrogenase type 2 (HSD2). The HSD2 enzyme catalyzes the oxidation of receptor active endogenous glucocorticoids (cortisol in humans, corticosterone in rodents) to their biologically inert metabolites (cortisone, 11-dehydrocorticosterone), while synthetic glucocorticoids such as dexamethasone tend to cross the placenta in their active form [5]. The role of placental HSD2 enzyme activity in protecting the fetus from maternal glucocorticoid overexposure has been highlighted in both human and animal studies. Decreased birth weight and a variety of programmed phenotypes have been linked to mutation or genetic knock-out of the HSD2 gene, the pharmacological inhibition of HSD2 enzyme activity, or the administration of synthetic glucocorticoids, which are not actively oxidized by HSD2 [6].

Previous studies in rats have shown that administration of a low-protein diet throughout pregnancy results in a decrease in placental HSD2 expression leading to low birth weight and a hypertensive phenotype in the offspring [4, 7]. Interestingly, even when pregnant dams are placed on the low-protein diet for discrete periods lasting only 7 days (i.e., days 0–7, 8–14, or 15–22), the offspring exhibit signs of hypertension, albeit to a lesser degree than offspring of dams who consumed the diet throughout pregnancy [8]. Furthermore, administration of a low-protein diet during the final week of pregnancy resulted in a more severe phenotype in the offspring than administration of the diet during early or mid-gestation [8]. The administration of a synthetic glucocorticoid to pregnant rats in the final week of gestation has also been shown to program hypertension in the offspring [9]. 

We have recently demonstrated that a low-protein diet results in low birth weight and deficits in cardiovascular function in mice [10, 11]. We have also shown that dexamethasone (a poor substrate for HSD2 metabolism) administration to mice during the second half of pregnancy induces cardiovascular dysfunction [11]. Together, these data suggest that, as in rats, mice can be programmed for adult cardiovascular disease by both dietary manipulation and direct exposure to glucocorticoids during fetal life, and as such, the latter stages of gestation may represent a critical window in which fetal glucocorticoid exposure must be tightly regulated. 

To date, the majority of data available concerning placental glucocorticoid metabolism and fetal programming via maternal dietary manipulation is derived from studies performed in rats. Given the array of genetic tools currently available for use in mice, a better understanding of normal and pathophysiological processes in this important animal model is of critical importance. Additionally, nothing is known about the sex-specific expression of placental HSD2 expression in mice. The goal of the current study was to examine the effects of a maternal low-protein diet on placental HSD2 protein and enzyme activity levels in C57BL/6 mice during late gestation. 

## 2. Materials and Methods

### 2.1. Animals

 Adult C57BL/6 mice (Jackson Laboratory, Bay Harbor, ME, USA) were maintained and bred at the University of Iowa Animal Care Unit. Prior to experimentation, animals were allowed access to standard mouse chow and water *ad libitum*. For breeding, female mice were paired with a male for up to 5 days and monitored for the presence of a vaginal plug each morning, an indicator of mating. Mice possessing a vaginal plug (embryonic day 0, E0) were removed from the cages and placed on either a normal protein diet (18% protein) or an isocaloric, low-protein diet (9% protein) for the duration of pregnancy as previously described [10]. Isocaloric 18% protein (cat. # 111269) and 9% protein rodent diets (cat. # 111270) were purchased from Dyets, Inc. (Bethlehem, PA, USA).

### 2.2. Tissue Collection

 On embryonic days 9.5, 12.5, 14.5 or 18.5, pregnant females were rendered unconscious via CO_2_ exposure and euthanized via cervical dislocation. Following removal of the uterus, uterine horns were incised and the pups and entire placentas were removed. Placentas were dissected from the pups and minced into several pieces. Typically, tissue samples from 4-5 placentas per litter were combined in sterile microfuge tubes and quickly frozen on dry ice for later use. 

### 2.3. Immunoblot Analysis

 Placental tissues were homogenized on ice in tissue lysis buffer (Promega, Madison, WI, USA) supplemented with protease inhibitor cocktail (Roche Applied Science, Indianapolis, IN). Samples were centrifuged for 10 minutes at 10,000 ×g to pellet cellular debris and the supernatant isolated. Supernatant protein concentrations were determined using the Bio-Rad Protein Assay according to the manufacturer's instructions (Bio-Rad, Hercules, CA, USA). Fifty micrograms of placental homogenate protein, or 20 *μ*g of mouse kidney protein (a positive control for immunoreactive HSD2 protein), were subjected to one-dimensional sodium dodecyl sulfate polyacrylamide gel electrophoresis (SDS-PAGE, 10% Tris-HCl gel) and transferred to nitrocellulose membrane. Nonspecific binding sites were blocked by incubating the membranes in Odyssey Blocking Buffer (Licor Biosciences, Lincoln, NE, USA). Blotting was performed overnight at 4°C using rabbit anti-mouse HSD2 primary antibody at a concentration of 2 *μ*g/mL (Alpha Diagnostics International, San Antonio, TX, USA). The membranes were washed extensively in PBS supplemented with 0.1% Tween-20, then incubated with IRDye anti-rabbit secondary antibody, and imaged using the Odyssey Infrared Imaging System (Licor Biosciences, Lincoln, NE, USA). Blots were then stripped in 0.2% NaOH for 5 minutes followed by incubation with goat anti-mouse *β*-actin primary antibody (Alpha Diagnostics International, San Antonio, TX, USA) at a concentration of 1 *μ*g/mL for one hour at room temperature. The membranes were then washed and incubated with IRDye anti-goat secondary antibody and imaged and immunoreactive bands quantified using the Odyssey Infrared Imaging System. HSD2 protein levels were normalized to those of *β*-actin on stripped and reprobed membranes to control for any variations in loading.

### 2.4. HSD2 Enzyme Activity Assay

 Placental tissues were homogenized in assay buffer (phosphate buffered saline supplemented with 250 mM sucrose) on ice and centrifuged for 10 minutes at 10,000 ×g to pellet cellular debris and the supernatant isolated. Supernatant protein concentrations were assayed as described above. For enzyme activity assays, 100 *μ*g of placental protein were combined with 1 mM NAD^+^ and 1 nM ^3^[H]-corticosterone and incubated at 37°C for 30 minutes. Tritiated steroid metabolites were isolated, separated via thin layer chromatography, and quantified as previously described [12]. HSD2 enzyme activity was quantified by assessing the conversion of tritiated corticosterone to 11-dehydrocorticosterone. 

### 2.5. DNA Isolation and PCR Analyses

Total DNA was isolated from tail snips taken from individual fetal mice harvested on day E18.5 of gestation using the DNeasy Blood and Tissue Kit according to the manufacturer's directions (Qiagen Inc., Valencia, CA, USA). Polymerase chain reaction was performed to amplify a homologous region of the X and Y chromosomes encoding the *Jarid1c* (X chromosome) and *Jarid1d* genes (Y chromosome) as previously described [13]. Briefly, 1 *μ*g of total DNA was combined with 45 *μ*L of Platinum PCR SuperMix (Invitrogen, Carlsbad, CA, USA) and 0.5 *μ*M of each primer (FWD: CTGAAGCTTTTGGCTTTGAG, REV: CCACTGCCAAATTCTTTGG) in a total reaction volume of 50 *μ*L. Following a 5-minute incubation at 95°C, thermal cycling was performed for 35 cycles (95°C for 30 seconds, 49°C for 60 seconds, and 72°C for 40 seconds), followed by a final extension at 72°C for 10 minutes. PCR products were separated on a 2% agarose gel and stained with ethidium bromide.

## 3. Results

### 3.1. Placental HSD2 Protein Expression

**Figure 1:**
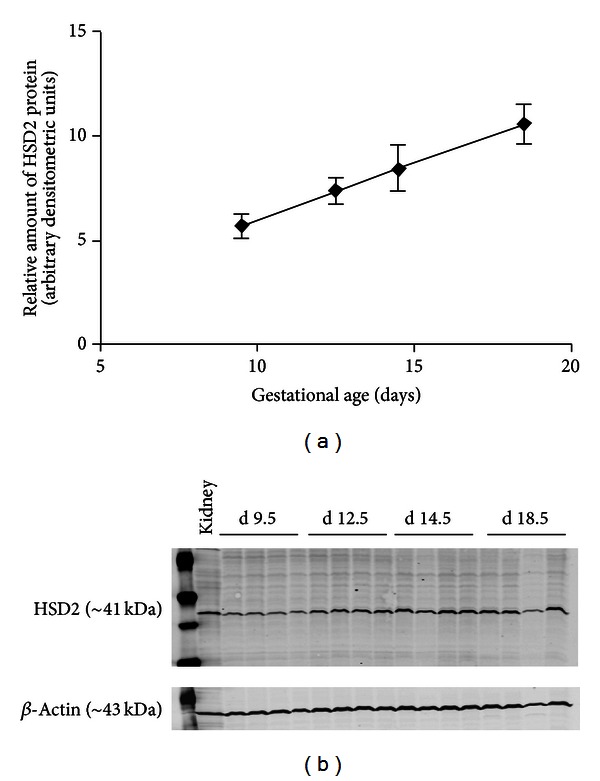
Placental HSD2 protein expression during the last half of gestation from pooled placentae of C57BL/6 mice (*n* = 4 litters at each time point). Data are expressed as arbitrary densitometric units normalized to *β*-actin loading control. A representative HSD2 immunoblot is shown in the figure.

**Figure 2:**
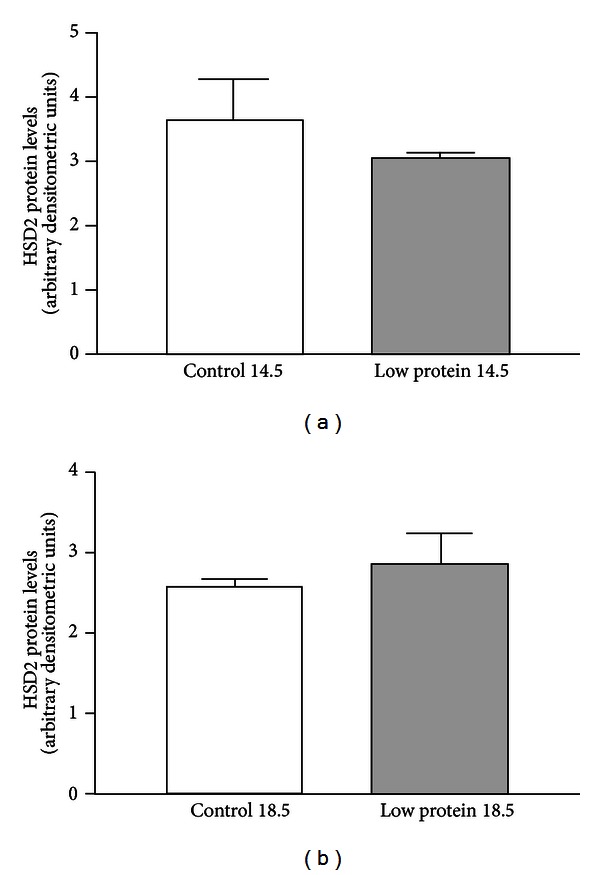
Placental HSD2 protein expression in pooled placentae harvested from mice consuming a control (18% protein) or low-protein (9% protein) diet during late gestation. Data are expressed as arbitrary densitometric units normalized to *β*-actin loading controls. (a) summarizes HSD2 protein expression at day E14.5. (b) summarizes HSD2 protein expression at day E18.5. *N* = 3 pooled litters for each group.

In order to assess the effects of maternal low-protein diet on placental HSD2 protein expression in mice, pregnant C57BL/6 mice were fed either a control diet (18% protein) or an isocaloric low-protein diet (9% protein) beginning on day E0 of gestation. Immunoblot analysis of placental tissues harvested from control mice on days E9.5, E12.5, E14.5, and E18.5 revealed a gradual accumulation of HSD2 protein towards the end of gestation (Figure 1). After characterizing the expression of HSD2 protein in control mice, we next compared the expression of this protein in late gestation placentas from mice who were fed either an 18% protein diet or an isocaloric 9% protein diet. As shown in Figure 2 (expressed as arbitrary densitometric units), the expression of HSD2 protein was not significantly (via unpaired Student's *t*-test) different in the placentas of the two groups at either days E14.5 (3.645 ± 0.645 versus 3.050 ± 0.080) or E18.5 (2.600 ± 0.100 versus 2.873 ± 0.383).

### 3.2. Placental HSD2 Enzyme Activity

**Figure 3:**
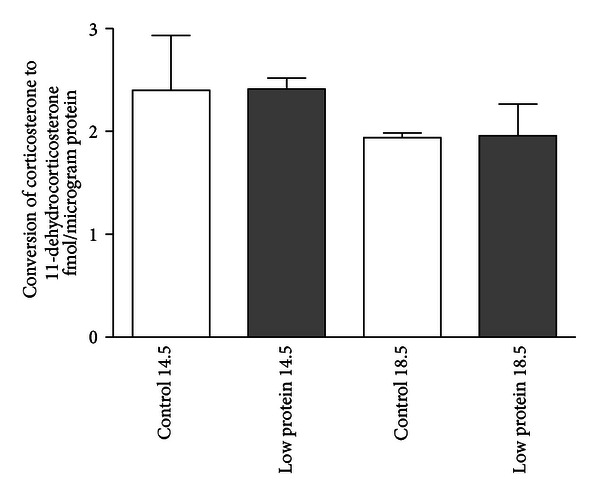
Placental HSD2 enzyme activity in pooled placentae. Data are expressed as femtomoles of 11-dehydrocorticosterone produced per microgram of placental protein. *N* = 3 litters for each group.

Previous studies have demonstrated that HSD2 enzyme activity may not be directly related to actual HSD2 protein levels due to posttranslational modifications or chemical inhibition. Indeed, decreases in HSD2 enzyme activity have been observed under conditions where HSD2 protein levels remain unchanged [14–16]. Therefore, we sought to explore the possibility that maternal low-protein diet induces changes in HSD2 enzyme activity in the absence of changes in actual HSD2 protein levels. To address this, enzyme activity assays were performed on pooled placental tissues harvested from control and low-protein mouse litters (3-4  litters, 5–7 pups per litter) on days E14.5 and E18.5. As shown in Figure 3, HSD2 enzyme activity (expressed as femtomoles of 11-dehydrocorticosterone produced per microgram of placental protein) was virtually identical in placental tissue samples harvested from mice on both days E14.5 and E18.5, irrespective of the diet consumed during gestation (2.402 ± 0.531 versus 2.410 ± 0.109 on E14.5; 1.941 ± 0.045 versus 1.959 ± 0.308 on E18.5). 

### 3.3. Effects of Gender on Placental HSD2 Levels

**Figure 4:**
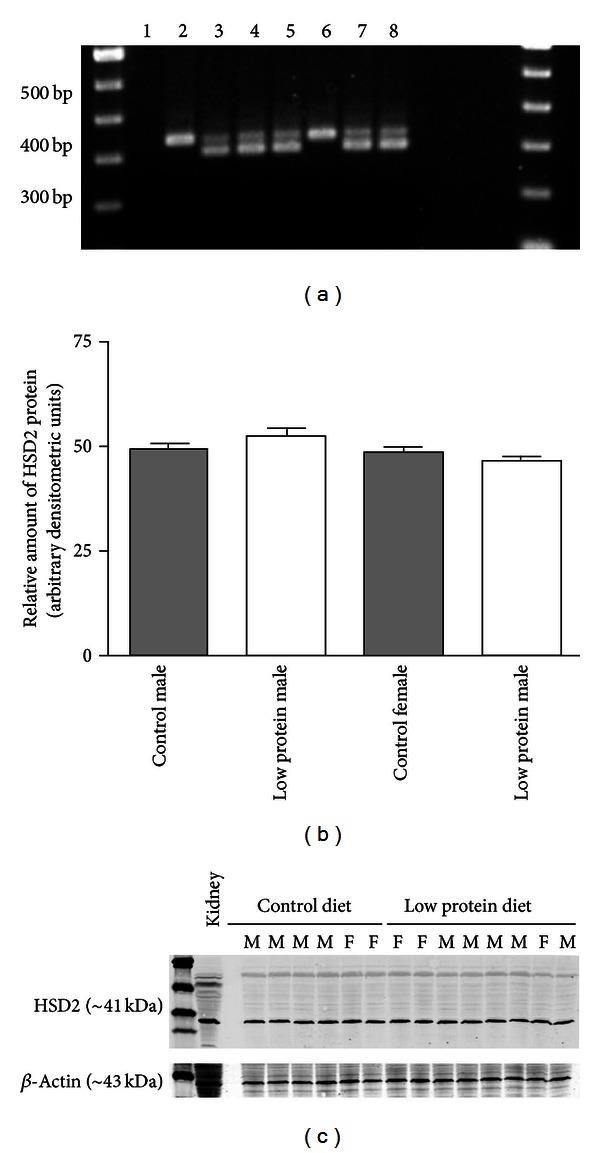
Gender-specific placental expression of HSD2 protein during late gestation (E18.5). (a) shows representative agarose gel of PCR products used to identify gender of each placenta. Lane 1 is a water only negative control. Male mice exhibited two PCR products, one amplified from the X chromosome and one from the Y chromosome. Females exhibited only one PCR product amplified from the X chromosome. (b) shows summary of placental HSD2 protein levels in male and female placentae from each treatment group. Data are expressed as arbitrary densitometric units normalized to *β*-actin loading control. A representative immunoblot is shown in (c). *N* = 3–5 placentae each from at least two separate litters.

Although HSD2 protein and enzyme activity levels in late gestation pooled placentas were not significantly affected by maternal low-protein diet, we sought to determine if there was a link between HSD2 protein expression and the gender of the offspring. Sex-specific differences in placental HSD2 activity have been reported in some human pregnancies and the effect of low-protein diets on adult phenotype in rodents differ markedly by gender [10, 17, 18]. To explore the possible role of gender in placental HSD2 expression, individual placentas were harvested on day E18.5 from pregnant mice fed either a control or low-protein diet. DNA isolated from tail snips of the corresponding fetuses was used in PCR reactions to determine the gender of each offspring (Figure 4(a)). As shown in Figure 4(b), on day E18.5, HSD2 protein levels in each placenta were nearly identical irrespective of gender or maternal diet during gestation. 

## 4. Discussion

The concept of the fetal programming of adult disease is an area of intense current research and its complexity is confounded by findings that multiple causative events are likely at play. Several lines of data suggest that events occurring *in utero* can have long-term, deleterious effects on the endocrine, metabolic, cardiovascular, and neurological physiology of the offspring, often in a sex-specific manner. Studies in a variety of animal models have shown that a “programmed” phenotype can be induced by numerous events including maternal dietary intervention (global caloric restriction, high-fat diet, low-protein diet, etc.), induction of maternal psychological stress, intrauterine growth restriction, among others [19–21]. Furthermore, strong links have been drawn between fetal programming, the reduced activity of placental HSD2, and glucocorticoid overexposure *in utero, *especially in late gestation [19, 22]. In rats, the consumption of a low-protein diet by pregnant dams has been shown to reduce the placental expression of HSD2 during late gestation and result in vascular defects and hypertension in the offspring [7, 23]. 

Currently, the vast majority of published data concerning placental HSD2 expression and activity, as it relates to fetal glucocorticoid overexposure and fetal programming, has come from studies in humans, rats, and sheep. Little information has been published on the expression and regulation of placental HSD2 in mouse models, despite this animal model's widespread use in the study of cardiovascular and metabolic physiology and its unique capacity for genetic manipulation. In an attempt to address this deficit, we examined the expression and enzyme activity of HSD2 in late gestation placentas of C57BL/6 mice from mothers consuming a protein-restricted diet throughout pregnancy. 

Previous studies in rats have demonstrated that a maternal low-protein diet (isocaloric 18% versus 9% protein) is sufficient to trigger a reduction in the placental expression of HSD2 concomitant with decreased birth weight, decreased renal expression of HSD2, altered vascular reactivity, and hypertension in the offspring [7, 23]. These studies, and others, suggest a paradigm by which a reduction in placental HSD2 leads to increased passage of maternal glucocorticoids to the fetal circulation and the resultant “programmed” phenotype. In support of this, numerous studies have shown that maternal administration of synthetic glucocorticoids, such as dexamethasone, is sufficient to elicit a “programmed” phenotype in rats and sheep [24–26]. Recent studies in this lab have shown that a similar dietary program during pregnancy leads to decreased birth weight, altered vascular reactivity, and hypertension in male mice, similar to what has been observed in rats [10, 11]. However, as shown in Figures 2 and 3, the expression of HSD2 protein and actual enzyme activity in the placentas of mice fed a low-protein diet did not differ significantly from controls during late gestation (E14.5 and E18.5) and was further confirmed by sex-specific analysis of placental HSD2 expression on day E18.5 (Figure 4). The lack of effect of the low-protein diet on placental HSD2 protein levels was initially unexpected but is not entirely dissimilar to another recent report in mice where researchers observed only a modest effect of a low-protein diet on placental HSD2 enzyme activity and also suggest that placental HSD2 may only play a minor, if any, role in mediating these fetal programming effects in mice [27]. Additionally, in C57BL/6 mouse placentae we observed a gradual increase in HSD2 protein levels, while Cottrell et al. observed a gradual decrease in HSD2 enzyme activity with advancing gestational age [27]. This apparent discrepancy may suggest that HSD2 protein expression and actual enzyme activity may be differentially regulated via posttranslational effects on the HSD2 protein, as we have previously shown [14]. Although we did not observe the same modest decrease in placental HSD2 enzyme activity in the low-protein group as these authors, this may be attributable to differences in methodologies employed. Notably, the authors of this study attempted to examine the activity of HSD2 only in the labyrinth zone of the placenta via blunt dissection of harvested tissues. In our study, we examined HSD2 enzyme activity in the entire placental mass. Although the labyrinth zone of the placenta exhibits the highest level of HSD2 gene expression, it should be noted that HSD2 is not entirely restricted to this zone [28]. Furthermore, our “low-protein” diet contained 9% casein protein versus the slightly lower 8% casein in the study by Cottrell et al. [27]. Finally, our current study carried the analysis further by examining specific placental HSD2 levels in placentae from male versus female mouse offspring in late gestation (Figure 4). 

Given the somewhat unexpected results of our study and those of the recent study by Cottrell et al. [27], it appears likely that fetal programming of mice, via maternal dietary manipulation, may be mediated by pathways or events that are distinct from those in other commonly studied other animal models (i.e., downregulation of placental HSD2). Indeed, this study demonstrated that maternal protein restriction in mice resulted in a precocious activation of the fetal hypothalamic-pituitary-adrenal (HPA) axis, suggesting the possibility that in mice, maternal protein restriction triggers the “programmed” phenotype not by fetal overexposure to maternal glucocorticoids, but by the overproduction of fetal glucocorticoids.

While precocious activation of the fetal HPA axis could contribute to fetal glucocorticoid overexposure in the low-protein diet model, it may be unwise to completely rule out the role of maternal glucocorticoid transfer across the placenta. The ATP-cassette binding protein (Abcb1), p-glycoprotein, is a broad spectrum drug efflux pump that is known to transport glucocorticoids out of a wide variety of cells and has been shown to inhibit the transfer of a variety of substrates, including glucocorticoids, from the maternal circulation to the fetal circulation [29–31]. P-glycoprotein is expressed in the mouse placenta throughout gestation and likely plays a protective role in preventing the transfer of maternal glucocorticoids to the fetus [30]. It is currently unknown whether the expression and activity of placental p-glycoprotein is influenced by maternal protein restriction. Future studies aimed at examining the expression and activity of placental p-glycoprotein in a low-protein diet animal model will offer further insight into the mechanisms by which glucocorticoids influence fetal programming. 

## 5. Conclusions

The role of placental glucocorticoid metabolism in the pathogenesis of fetal programming of adult onset disease continues to be an area of active research. Deficits in placental HSD2 gene expression and metabolism as a consequence of maternal protein restriction have been observed in several species including rats. Despite its widespread use in biomedical research, little is known about this phenomenon in common laboratory mouse strains, nor whether the expression of placental HSD2 is influenced by gender of the offspring. In the current study, we observed that placental HSD2 gene expression and enzyme activity was unaffected by maternal protein restriction (18% protein versus 9% protein) in C57BL/6 mice. These findings are in contrast to observations in other animal models (i.e., rats), but are in general agreement with another recent study in mice [27]. Furthermore, we observed that HSD2 expression and activity was similar in male and female placentae and that this was also not influenced by maternal diet. These results, while initially unexpected, represent some of the first data concerning the effects of maternal diet on placental HSD2 expression in mice and the first data examining the effect of gender on the expression and activity of this placental enzyme. Despite previous studies demonstrating a clear “programmed” adult phenotype in response to maternal protein restriction in this mouse strain, it appears that this effect is not mediated by changes in placental glucocorticoid metabolism as it is in other animal models. 

## References

[1] McMillen IC, Robinson JS (2005). Developmental origins of the metabolic syndrome: prediction, plasticity, and programming. *Physiological Reviews*.

[2] Langley-Evans SC (2001). Fetal programming of cardiovascular function through exposure to maternal undernutrition. *Proceedings of the Nutrition Society*.

[3] Meaney MJ, Szyf M, Seckl JR (2007). Epigenetic mechanisms of perinatal programming of hypothalamic-pituitary-adrenal function and health. *Trends in Molecular Medicine*.

[4] Langley-Evans SC, Phillips GJ, Benediktsson R (1996). Protein intake in pregnancy, placental glucocorticoid metabolism and the programming of hypertension in the rat. *Placenta*.

[5] Krozowski Z, Li KXZ, Koyama K (1999). The type I and type II 11*β*-hydroxysteroid dehydrogenase enzymes. *Journal of Steroid Biochemistry and Molecular Biology*.

[6] Seckl JR (2004). Prenatal glucocorticoids and long-term programming. *European Journal of Endocrinology, Supplement*.

[7] Bertram C, Trowern AR, Copin N, Jackson AA, Whorwood CB (2001). The maternal diet during pregnancy programs altered expression of the glucocorticoid receptor and type 2 11*β*-hydroxysteroid dehydrogenase: potential molecular mechanisms underlying the programming of hypertension in utero. *Endocrinology*.

[8] Langley-Evans SC, Welham SJM, Sherman RC, Jackson AA (1996). Weanling rats exposed to maternal low-protein diets during discrete periods of gestation exhibit differing severity of hypertension. *Clinical Science*.

[9] Levitt NS, Lindsay RS, Holmes MC, Seckl JR (1996). Dexamethasone in the last week of pregnancy attenuates hippocampal glucocorticoid receptor gene expression and elevates blood pressure in the adult offspring in the rat. *Neuroendocrinology*.

[10] Roghair RD, Segar JL, Volk KA (2009). Vascular nitric oxide and superoxide anion contribute to sex-specific programmed cardiovascular physiology in mice. *American Journal of Physiology*.

[11] Roghair RD, Segar JL, Kilpatrick RA, Segar EM, Scholz TD, Lamb FS (2007). Murine aortic reactivity is programmed equally by maternal low protein diet or late gestation dexamethasone. *Journal of Maternal-Fetal and Neonatal Medicine*.

[12] Garbrecht MR, Klein JM, McCarthy TA, Schmidt TJ, Krozowski ZS, Snyder JM (2007). 11-*β* Hydroxysteroid dehydrogenase type 2 in human adult and fetal lung and its regulation by sex steroids. *Pediatric Research*.

[13] Clapcote SJ, Roder JC (2005). Simplex PCR assay for sex determination in mice. *BioTechniques*.

[14] Garbrecht MR, Krozowski ZS, Snyder JM, Schmidt TJ (2006). Reduction of glucocorticoid receptor ligand binding by the 11-*β* hydroxysteroid dehydrogenase type 2 inhibitor, Thiram. *Steroids*.

[15] Atanasov AG, Tam S, Röcken JM, Baker ME, Odermatt A (2003). Inhibition of 11*β*-hydroxysteroid dehydrogenase type 2 by dithiocarbamates. *Biochemical and Biophysical Research Communications*.

[16] Gomez-Sanchez EP, Ganjam V, Chen YJ, Liu Y, Clark SA, Gomez-Sanchez CE (2001). The 11*β* hydroxysteroid dehydrogenase 2 exists as an inactive dimer. *Steroids*.

[17] Murphy VE, Gibson PG, Giles WB (2003). Maternal asthma is associated with reduced female fetal growth. *American Journal of Respiratory and Critical Care Medicine*.

[18] Moritz KM, Cuffe JSM, Wilson LB (2010). Review: sex specific programming: a critical role for the renal renin-angiotensin system. *Placenta*.

[19] O'Donnell KJ, Bugge Jensen A, Freeman L, Khalife N, O'Connor TG, Glover V (2012). Maternal prenatal anxiety and downregulation of placental 11*β*-HSD2. *Psychoneuroendocrinology*.

[20] Langley-Evans SC (2001). Fetal programming of cardiovascular function through exposure to maternal undernutrition. *Proceedings of the Nutrition Society*.

[21] Knight BS, Pennell CE, Shah R, Lye SJ (2007). Strain differences in the impact of dietary restriction on fetal growth and pregnancy in mice. *Reproductive Sciences*.

[22] Seckl JR, Holmes MC (2007). Mechanisms of disease: glucocorticoids, their placental metabolism and fetal “programming” of adult pathophysiology. *Nature Clinical Practice Endocrinology and Metabolism*.

[23] Brawley L, Itoh S, Torrens C (2003). Dietary protein restriction in pregnancy induces hypertension and vascular defects in rat male offspring. *Pediatric Research*.

[24] Nyirenda MJ, Lindsay RS, Kenyon CJ, Burchell A, Seckl JR (1998). Glucocorticoid exposure in late gestation permanently programs rat hepatic phosphoenolpyruvate carboxykinase and glucocorticoid receptor expression and causes glucose intolerance in adult offspring. *Journal of Clinical Investigation*.

[25] Long NM, Shasa DR, Ford SP, Nathanielsz PW (2012). Growth and insulin dynamics in two generations of female offspring of mothers receiving a single course of synthetic glucocorticoids. *American Journal of Obstetrics and Gynecology*.

[26] Roghair RD, Lamb FS, Miller FJ, Scholz TD, Segar JL (2005). Early gestation dexamethasone programs enhanced postnatal ovine coronary artery vascular reactivity. *American Journal of Physiology*.

[27] Cottrell EC, Holmes MC, Livingstone DE, Kenyon CJ, Seckl JR (2012). Reconciling the nutritional and glucocorticoid hypotheses of fetal programming. *FASEB Journal*.

[28] Thompson A, Han VKM, Yang K (2002). Spatial and temporal patterns of expression of 11*β*-hydroxysteroid dehydrogenase types 1 and 2 messenger RNA and glucocorticoid receptor protein in the murine placenta and uterus during late pregnancy. *Biology of Reproduction*.

[29] Behravan J, Piquette-Miller M (2007). Drug transport across the placenta, role of the ABC drug efflux transporters. *Expert Opinion on Drug Metabolism and Toxicology*.

[30] Kalabis GM, Kostaki A, Andrews MH, Petropoulos S, Gibb W, Matthews SG (2005). Multidrug resistance phosphoglycoprotein (ABCB1) in the mouse placenta: fetal protection. *Biology of Reproduction*.

[31] Parry S, Zhang J (2007). Multidrug resistance proteins affect drug transmission across the placenta. *American Journal of Obstetrics and Gynecology*.

